# Harnessing Friction in Intertwined Structures for High‐Capacity Reusable Energy‐Absorbing Architected Materials

**DOI:** 10.1002/advs.202105769

**Published:** 2022-03-08

**Authors:** Jinyou Li, Zhe Chen, Qunyang Li, Lihua Jin, Zhihua Zhao

**Affiliations:** ^1^ School of Aerospace Engineering Tsinghua University Beijing 100084 P. R. China; ^2^ Department of Mechanical and Aerospace Engineering University of California, Los Angeles Los Angeles CA 90095 USA

**Keywords:** architected materials, energy dissipation, energy‐absorbing materials, friction, intertwined structure, porous structure

## Abstract

Energy‐absorbing materials with both high absorption capacity and high reusability are ideal candidates for impact protection. Despite great demands, the current designs either exhibit limited energy‐absorption capacities or perform well only for one‐time usage. Here a new kind of energy‐absorbing architected materials is created with both high absorption capacity and superior reusability, reaching 10 kJ kg^−1^ per cycle for more than 200 cycles, that is, unprecedentedly 2000 kJ kg^−1^ per lifetime. The extraordinary performance is achieved by exploiting the rate‐dependent frictional dissipation between prestressed stiff cores and a porous soft elastomer, which is reinforced by an intertwined stiff porous frame. The vast interfaces between the cores and elastomer enable high energy dissipation, while the magnitude of the friction force can adapt passively with the loading rate. The intertwined structure prevents stress concentration and ensures no damage and reusability of the constituents after hundreds of loading cycles. The behaviors of the architected materials, such as self‐recoverability, force magnitude, and working stroke, are further tailored by tuning their structure and geometry. This design strategy opens an avenue for developing high‐performance reusable energy‐absorbing materials that enable novel designs of machines or structures.

## Graphical abstract

In current energy‐absorbing materials, capacity and reusability seem to be mutually exclusive properties. Here a new kind of architected materials is created to obtain both high energy‐absorbing capacity and high reusability. The extraordinary performance is achieved by exploiting frictional dissipation within an intertwined structure, composed of porous soft elastomer, porous stiff frame, and sliding cores.

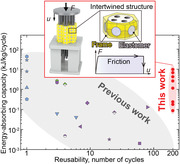

## 1 Introduction

Energy‐absorbing materials and structures,^[^ ^1^ ^]^ such as foams in helmets and car bumpers, play an important role in keeping humans and objects safe from unexpected impacts.^[^ ^2^, ^3^ ^]^ This protective functionality demands two iconic features of their force–displacement relations: hysteresis and limited peak force.^[^ ^4^ ^]^ They ensure that impact energy is dissipated without imposing high stress onto protected targets. Hence, an ideal force–displacement curve of an energy‐absorbing material is rectangular‐shaped and has a long and flat force plateau. Achieving such a curve is crucial for creating exceptional energy‐absorbing materials, and has attracted great research interest in exploring original design strategies.^[^ ^5^, ^6^ ^]^

The most well‐known energy‐absorbing mechanism is damaging constituent materials, such as ductile metals,^[^ ^7^, ^8^ ^]^ brittle foams,^[^ ^9^, ^10^, ^11^ ^]^ and ceramics.^[^ ^12^ ^]^ Besides, in order to maintain a long yielding force plateau, curved shapes^[^ ^13^, ^14^ ^]^ or auxetic materials^[^ ^15^ ^]^ are introduced to prevent structures from immediately losing their load‐carrying capabilities due to localization. The mechanisms of damage and plastic flow can dissipate a huge amount of energy, benefited from bond breakage or dislocations motion at the molecule level. Taking commercial aluminum foams as an example,^[^ ^16^, ^17^ ^]^ their energy‐absorbing capacities are as high as 30 kJ kg^−1^ or 30 MJ m^−3^. The excellent performance has endowed them with broad applications in engineering, such as protecting cargo^[^ ^18^ ^]^ and preventing collapse of rocks in mining.^[^ ^19^ ^]^ However, they are usually for one‐time usage, after which the constituents are permanently damaged. This shortcoming can be partially overcome by incorporating damage‐tolerant micro‐lattices^[^ ^20^, ^21^, ^22^, ^23^, ^24^, ^25^ ^]^ or phase‐transforming constituents^[^ ^26^, ^27^, ^28^ ^]^ that allow the materials to undergo cyclic loadings, although the performance decreases along cycles.

To completely overcome the one‐time‐usage limitation, a promising way is designing the microstructures of architected metamaterials, which provides a vast space to gain outstanding mechanical properties that are otherwise hard to achieve.^[^ ^29^ ^]^ By introducing non‐damage energy dissipation mechanisms into microstructures, researchers have developed reusable energy‐absorbing metamaterials. A well‐investigated mechanism is mechanical instability of micro‐cells, such as buckling of flexible beams^[^ ^30^, ^31^, ^32^ ^]^ and shells,^[^ ^33^ ^]^ and nonlinear forces between magnets.^[^ ^34^, ^35^ ^]^ The assembled structures, obtained by connecting a series of these micro‐cells, often produce hysteric saw‐tooth force–displacement curves.^[^ ^36^, ^37^ ^]^ The metamaterials constructed in this way are reusable since the deformation is elastic. Nevertheless, their energy‐absorbing capabilities are typically several orders of magnitude lower than those of the non‐reusable ones, which significantly limits their potential applications. For example, one kind of micro‐beam based metamaterial^[^ ^38^ ^]^ only has an energy‐absorption capacity around 0.15 kJ kg^−1^ or 0.015 MJ m^−3^.

**Figure 1:**
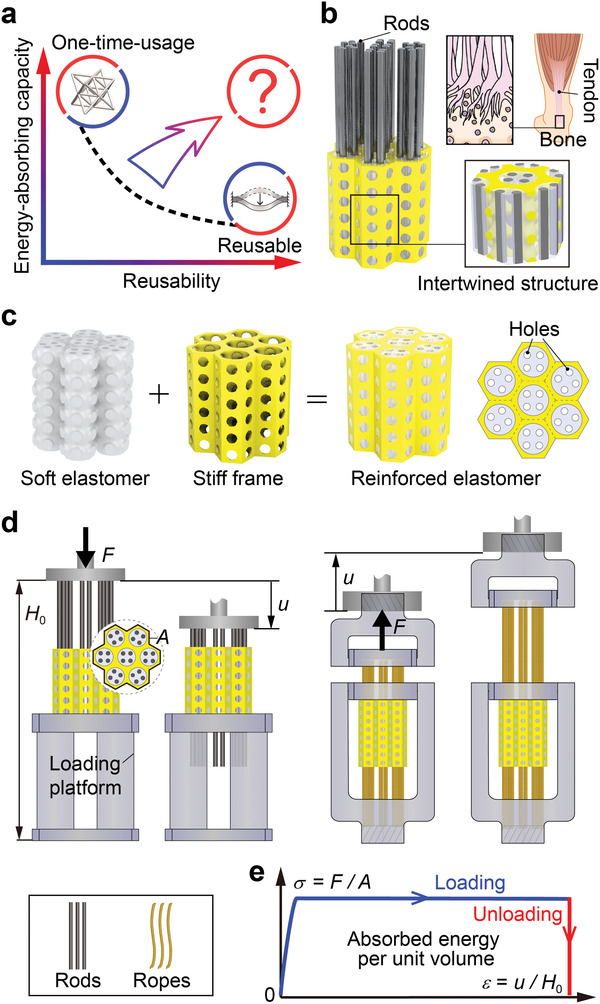
Design of the proposed energy‐absorbing architected materials with both high capacity and high reusability. a) A typical energy‐absorbing material shows a trade‐off between reusability and energy‐absorbing capacity. b,c) The proposed architected material is composed of stiff rods/ropes and a reinforced elastomer, which can slide between each other. The reinforced elastomer has a porous stiff frame intertwined with a porous soft silicone elastomer, inspired by tendon‐bone interfaces shown in upper right corner of (b). d) The architected material can be subjected to a compressive or tensile force *F*, producing a displacement *u*. The distance from the bottom of the loading platform to the top of the rods/ropes when displacement *u* = 0 is defined as height *H*_0_, and the cross‐sectional area of the reinforced elastomer in (c) is *A*. e) The measured force balances the friction forces between the rods/ropes and reinforced elastomer. The force *F* is expected to first sharply increase with the displacement *u* in the static friction region, and then reache a plateau in the dynamic friction region, which shows an ideal rectangular shape for energy absorption.

The relatively low performance of reusable energy‐absorbing metamaterials is mainly caused by two reasons: the constituent materials can only sustain limited forces, and only a small portion of the microstructures contribute to energy dissipation.^[^ ^39^ ^]^ A strategy to improve the capacity is increasing the peak forces of the saw‐teeth^[^ ^40^, ^41^ ^]^ through increasing the maximum stresses and using stiffer materials. However, this method endangers both the constituent materials and protected targets. Researchers recently attempted other non‐damage energy dissipation mechanisms, such as visco‐elasticity^[^ ^42^ ^]^ and dry friction^[^ ^43^, ^44^, ^45^ ^]^ between sliding particles, which have been utilized in some vibration dampers,^[^ ^46^, ^47^ ^]^ to build energy‐absorbing metamaterials. Nevertheless, their designs are still in infancy and the obtained energy‐absorption capacities are as low as other reusable ones. Therefore, it is still an open problem to simultaneously achieve high reusability and high capacity in energy‐absorbing materials (**Figure** **1**a).

Here, we survey the frictional mechanism in depth, and realize a new kind of architected materials with extremely high energy‐absorbing capacity and reusability. They have rectangular‐shaped load–displacement curves, and their energy‐absorbing capacities are comparable to the non‐reusable ones, reaching 0.1 − 10 kJ kg^−1^ or 0.1 − 13 MJ m^−3^. The basic idea is maximizing friction forces by maximizing the true contact area and normal stress at the frictional interfaces. Particularly, the contact area is enlarged by two strategies: selecting an elastomer as a constituent due to its ability of conformal contact to create a large true contact area at the micro‐scale, and designing an elastomer into a porous structure to involve a large interface within a limited volume. To create large normal stress, we introduce prestress between a soft porous elastomer and stiff rods/ropes inserted into it by geometric mismatches. Meanwhile, another stiff porous frame is intertwined with the elastomer to significantly enhance the rigidity of the structure and enlarge the prestress between the sliding parts (Figure 1b, c). The obtained load–displacement curve has a long plateau (Figure 1d, e), whose height and length can be easily tuned by tailoring the geometry. Moreover, the energy‐absorbing capacity shows no sign of notable decreasing after 200 loading–unloading cycles in experiments, indicating the outstanding reusability. An additional benefit of this design is passively adaptive energy‐absorbing ability under different impact velocities, which is sourced from the rate‐dependent friction coefficient between the elastomer and stiff rods/ropes and further magnified by geometric and material nonlinearity. This property is further utilized to create self‐recoverable architected materials. All these features shape the reported architected materials to be ideal candidates for energy absorption.

## 2 Results

### 2.1 Intertwined Structure and Energy‐Absorbing Performance

Figure 1b sketches the overall design of the proposed architected materials. We intertwine a soft porous silicone elastomer structure (gray) with a stiff porous frame (yellow) to obtain a reinforced elastomer that has a better load‐bearing capability. This design was inspired by human's tendon‐bone interface,^[^ ^48^, ^49^ ^]^ where soft tendon tissue interconnects with hard bone tissue to achieve a robust anchor (upper right part of Figure 1b). Our intertwined structure was geometrically modified from Schwarz primitive minimal surface,^[^ ^50^ ^]^ which divides the space into two intertwined sub‐domains for the stiff frame and the soft elastomer, respectively. Here, the cubic packing of the Schwarz surface was changed to a hexagonal packing, since it has a better strength than a square or triangular one^[^ ^51^ ^]^ due to higher connectivity. Besides, the modified minimal surface can guarantee a smooth interface between the frame and the elastomer,^[^ ^50^ ^]^ thus avoiding unnecessary stress concentrations. More details on the structural geometry are given in Figure S1, Supporting Information. The stiff frame was 3D printed by polylactic acid (PLA) filament, while the porous silicone elastomer was cast using a mold composed of several parts (see Section 4 and Figure S2, Supporting Information).

Each hexagonal column of the reinforced elastomer has several straight holes, and steel or carbon fiber rods were inserted to these holes. The diameter of the rods is slightly larger than that of the holes, which creates pressure between the elastomer and the rods along the frictional surfaces. When the rods are pushed downward (Figure 1d), a compression force *F* balances the friction forces. When *F* is low, there is no relative displacement between the rods and the elastomer due to static friction, and *F* linearly increases with the deformation of the elastomer. When *F* is high, relative sliding between the rods and the elastomer occurs, and *F* remains at a plateau corresponding to the dynamic friction force (Movie S1, Supporting Information). To quantify the energy‐absorbing capacity, the nominal strain ε of the proposed architected materials is defined as the displacement *u* divided by the initial height *H*_0_ (Figure 1d), and the nominal stress σ as the force *F* divided by the cross‐section area *A* of the reinforced elastomer. Then, the nominal stress–strain curve is expected as Figure 1e. The area underneath the hysteric curve represents the absorbed energy per unit volume, where the volume of the proposed material is defined as *AH*_0_ that includes the space occupied by the reinforced elastomer, the rods, and the loading platform used to hold the reinforced elastomer. In addition, replacing the rods with ropes (right part of Figure 1d) should yield similar frictional behavior against tensile loads.

**Figure 2:**
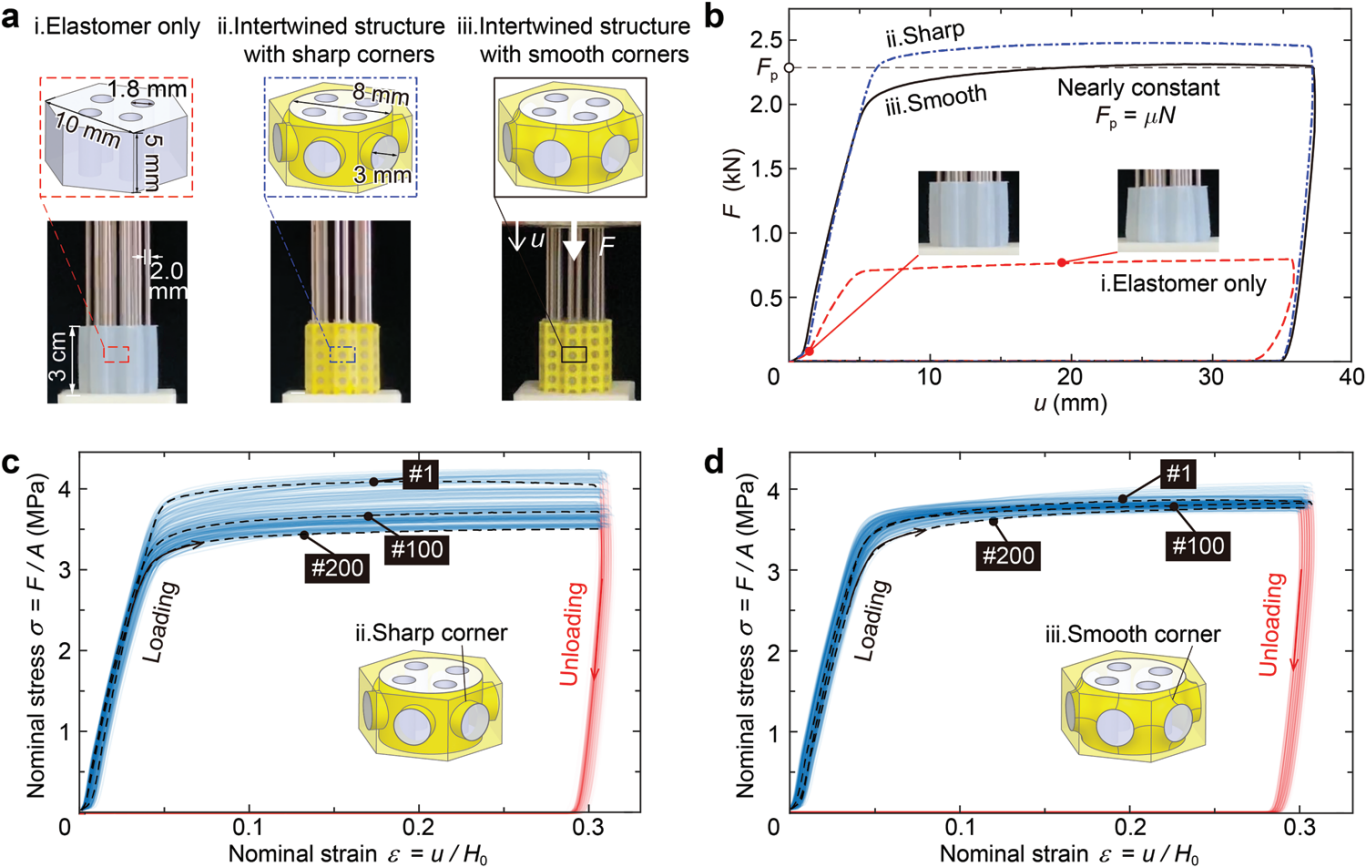
Experimental verification of the high capacity and reusability of the proposed energy‐absorbing architected materials. a) Comparison of three different structures, stacked up by cell (i), (ii) and (iii), to understand how soft elastomer and stiff frame affect the energy‐absorbing performance. The upper schematics display the structure of each cell. Cell (i) contains only an elastomer, (ii) and (iii) involve stiff frames with sharp and smooth corners, respectively, to reinforce the elastomer. Each cell has a height 5 mm and flat‐to‐flat distance 10 mm. The diameter of the rods 2.0 mm is slightly larger than that of the holes 1.8 mm to create pressure between the elastomer and the rods. The lower images show the corresponding manufactured seven‐column samples. b) Measured load–displacement curves for the structures in (a) with a compression loading rate *v* = 500 mm min^−1^. All the curves have nearly flat force plateaus *F*_p_ when the displacement is high. The two architected materials with intertwined structures exhibit higher *F*_p_, three times as many as that of the one with only an elastomer. Inset: images showing the elastomer without a stiff frame is more compliant and undergoes a larger deformation. c,d) All nominal stress–strain curves of structure (ii) and (iii) under 200 loading–unloading cycles in reusability tests. After 200 cycles, the capacity of the former decreases by about 14% , while that of the latter varies only about 4%.

As shown in **Figure** **2**a, we fabricated three different structures to reveal how the soft elastomer and stiff frame affect the energy‐absorbing performance. All the structures have seven columns, arranged as shown in Figure 1c, and each column is stacked up by six regular hexagonal cells of height 5 mm and flat‐to‐flat distance 10 mm. All the cells have four straight holes of diameter 1.8 mm. The differences among the cells of the three structures are: cell (i) is composed of an elastomer only; cell (ii) adopts a stiff porous frame, in which cylindrical bridges with sharp corners are used to connect adjacent cells, to reinforce the elastomer; and cell (iii) has bridges modified from Schwarz minimal surface to form smooth corners.

Inserting 2 mm‐diameter steel rods into the 1.8 mm‐diameter holes introduces prestress between the rods and the elastomer. Pushing rods downward, Figure 2b shows the obtained load–displacement curves, measured under displacement control by a single‐axis MTS Testing Machine with a loading rate *v* = 500 mm min^−1^. Each of these curves possesses a linearly increasing region, followed by a long plateau with a plateau force *F*_p_, just as expected. As a result, both structures (ii) and (iii) show a higher slope of the force–displacement curve in the linear region and a higher plateau force than those of structure (i) due to the reinforcement of the stiff frames. Besides, the plateau force *F*_p_ of structure (ii) is slightly larger than that of structure (iii), because the stiff frame of cell (ii) is thicker than cell (iii) and involves larger prestress. More quantitatively, the energy‐absorbing capacities of structure (ii) and (iii) are around 1.0 MJ m^−3^, which is about three times as large as that of structure (i) and comparable to non‐reusable energy‐absorbing materials, such as aluminum foams.

Further, we checked the reusability of structure (ii) and (iii) through cyclic loading. After 200 loading cycles at a loading rate of 500 mm min^−1^, the energy‐absorbing capacity of structure (ii) decreases by about 14% (Figure 2c), which might be caused by the stress concentration in the elastomer and potential debonding between the soft elastomer and stiff frame due to sharper corners. However, energy‐absorbing capacity of structure (iii) does not change notably (Figure 2d). Overall speaking, structure (iii) that modified from the minimal surface has better comprehensive performance on energy‐absorbing capacity and reusability, so that it is adopted here to design our architected materials.

**Figure 3:**
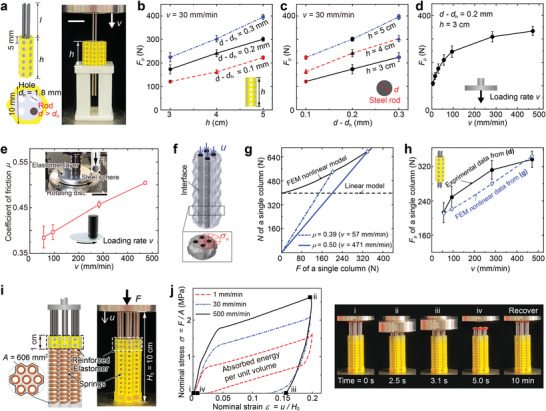
Dependence of *F*_p_ on the geometric parameters and loading rate *v*. a) Geometry of a single column of the reinforced elastomer (left), stacked up by multiple cells of type (iii) in Figure 2a. The image (right) shows the experimental setup to quantify the performance of a single column under certain loading rate *v*, in which the scale bar represents 2 cm. b–d) Dependence of *F*_p_ on *h*, *d* − *d*_h_, and *v*. The former two show linear relations, while the last one is nonlinear. For every data point, three samples were fabricated, and each sample was tested for four times; the upper and lower bounds of the the error bars represent the maxima and minima of the measured results. e) Measured μ − *v* curve between elastomer discs, with thickness 0.4 and 1.3 mm, and a steel sphere by the experimental setup shown in the inset, where the scale bar represents 2 cm. The upper and lower bounds of the error bars represent the maxima and minima of the results measured with different elastomer thickness. f) FEM model of a single column, whose stiff frame is set to be invisible for clarity. The rods are forced to move downward by displacement *u*, the vertical reaction force *F* is computed, and the normal force *N* is obtained by integrating the normal stress σ_*n*_ along the rods. g) *N* − *F* curve calculated from the FEM model in (f). h) Comparison of the experimentally measured *F*_p_ − *v* curve (black solid) and the numerically calculated *F*_p_ − *v* curve from the FEM model in (f,g). i,j) A self‐recoverable energy‐absorbing architected material, and its load–displacement curves under different loading rates. The right images show the configurations of the architected material corresponding to the different loading states indicated on the curve at a loading rate *v* = 500 mm min^−1^.

Next, we would like to understand how physical parameters affect energy‐absorbing capacity, which is mainly featured by *F*_p_ of a single column. Obviously, *F*_p_ can be tailored by varying the parameters, for example, the height *h* of the elastomer, or the diameter *d* of the steel rods (**Figure** **3**a). Theoretically, frictional dynamics says

(1) $$F_{p}=\mu N$$

where μ is the coefficient of dynamic friction between the elastomer and the rods, and *N* is the total normal contact force acting on the rods. Intuitively, increasing the column height *h* enlarges the contact area, and increasing the diameter difference between rods and holes *d* − *d*_h_ enlarges the prestress. As a result, *N*, and therefore *F*_p_, should be proportional to both *h* and *d* − *d*_h_, which was experimentally validated (Figure 3b,c). To simplify the experiments, only four steel rods were inserted into the central column for each seven‐column module (right in Figure 3a); thus the obtained plateau values *F*_p_ are about 1/7 of that in Figure 2b. Moreover, we observed *F*_p_ increases notably with the loading rate *v* (Figure 3d), which indicates that the energy‐absorbing capability gets higher for a higher impact velocity. This attractive feature will be quantified and understood in the next few paragraphs.

### 2.2 Mechanisms of Rate‐Dependent Energy‐Absorbing Behavior

The plateau force *F*_p_ = μ*N* relies on the loading rate *v*, indicating that at least one of μ and *N* changes with *v*. Further quantitative studies reveal they both are, which, more specifically, is attributed to the synergy of two iconic characteristics of elastomers: rate‐dependent frictional coefficient, and nonlinear hyperelastic property in large deformation.

Frictional coefficient μ of elastomers is rate‐dependent,^[^ ^52^ ^]^ which is closely related with viscoelastic property of elastomers.^[^ ^53^ ^]^ To quantify μ between our silicone elastomer and steel, we conducted friction experiments on a Tribometer (Rtec MFT‐V) (see details in Figure S3, Supporting Information). In the tribometer experiments, a steel sphere was pressed by a downward force *F*_*z*_ onto an elastomer disc, placed on a rigid flat plate (inset of Figure 3e). The plate was rotated to control the sliding speed, meanwhile, *F*_*z*_ was maintained to be an almost constant. Considering that μ relies on normal contact stress^[^ ^54^ ^]^ and elastomer thickness, we prepared two elastomer discs of thicknesses *t* = 0.4 and 1.3 mm, representing the thickness of the elastomer in the architected materials, which varies along the rod. The corresponding applied normal forces are *F*_*z*_ = 1.1 and 3.0 N respectively, to ensure the nominal normal contact stress acted on the steel sphere approaches the average normal stress applied to the rods, about 0.53 MPa according to the finite element method (FEM) simulations (for details see Figure S3b and Figure S4, Supporting Information), where the constitutive behavior of the silicone elastomer is assumed to follow the third order Ogden hyperelastic model^[^ ^55^ ^]^ (see Section 4 and Figure S5, Supporting Information). Taking the ratio of the lateral force to the normal force at different loading rates *v* gives us the frictional coefficient μ versus *v*. Averaging the measured data of the two elastomer samples yields the μ–*v* curve in Figure 3e. When *v* rises from 57 to 471 mm min^−1^, μ increases by 30%, while *F*_p_ = μ*N* increases by 60%, much greater than μ.

Therefore, besides μ, the normal contact force *N* at the steady sliding stage must also increase with *v*. We found this is caused by the combination of the geometric and material nonlinearity of the elastomer. It is well known that when a hyperelastic material is subject to a simple shear deformation, a normal stress needs to be applied, which is absent in linearly elastic materials.^[^ ^56^ ^]^ When the soft elastomer around the holes is subjected to frictional shear forces *F* from the rods, its large deformation tends to shrink the holes, which in turn enlarges *N*. This was validated via a FEM model of a column in the architected material subjected to a compressive force, as shown in Figure 3f (for details see Section 4 and Figure S4, Supporting Information). Rods and the elastomer were assumed to bond together. In quasi‐static numerical simulations, each rod had a diameter of 1.8 mm in the stress‐free state, and then was expanded to 2.0 mm to introduce prestress, after which the rods were forced to move downward by displacement *u*. Integrating normal stress over the interface gives the normal force *N*. The obtained *N* − *F* curve, black solid line in Figure 3g, confirms that *N* increases with *F*.

Combing the measured μ − *v* curve and the calculated *N* − *F* curve, we can quantitatively explain the rate‐dependent behavior *F*_p_ − *v* of the architected material. Under a given loading rate *v*, to reach the impending sliding condition, the normal and downward forces should satisfy *F* − μ(*v*)*N* = 0. Meanwhile, *F* and *N* are intrinsically related due to the geometric and material nonlinearity, as predicted by the FEM model. As shown in Figure 3g, under a given loading rate *v*, the linear relation *F* = μ(*v*)*N* intersects with the nonlinear *F* − *N* curve obtained by the FEM to determine the critical force *F*_p_(*v*) when impending sliding occurs. Several predicted values of *F*_p_ at different *v* are depicted by blue diamonds in Figure 3h; they agree well with the measured force data.

### 2.3 Self‐Recoverable Energy‐Absorbing Architected Materials

Here, the rate‐dependent behavior of the friction force was utilized to build a self‐recoverable energy‐absorbing architected material. As shown in Figure 3i, we integrate each column of the architected material with an off‐the‐shelf coil spring, whose stiffness is 4.85 N mm^−1^. When the rods are pushed downward, the springs are compressed; when the loading plate is retreated, forces in the spring return the rods back against friction forces. It is expected that the return rate decreases as the spring forces decrease. Therefore, when the return rate of the rods is less than the unloading rate of the testing machine *v*, the loading plate detaches from the rods and the compression force *F* drops to zero immediately (Figure 3j; Movie S2, Supporting Information). After detaching, the rods can be further pushed back by the springs since the resistant friction forces decrease at lower rates (right images in Figure 3j); this helps to extend the working stroke of the material.

The measured nominal stress–strain curves at different rates in Figure 3j confirm that a larger loading rate yields a larger hysteresis loop. At a loading rate of 500 mm min^−1^, the energy‐absorbing capacity is about 0.3 MJ m^−3^, which is about 20 times of that of the self‐recoverable energy‐absorbing metamaterials previously reported.^[^ ^38^ ^]^

### 2.4 Improving Energy‐Absorbing Capacity

**Figure 4:**
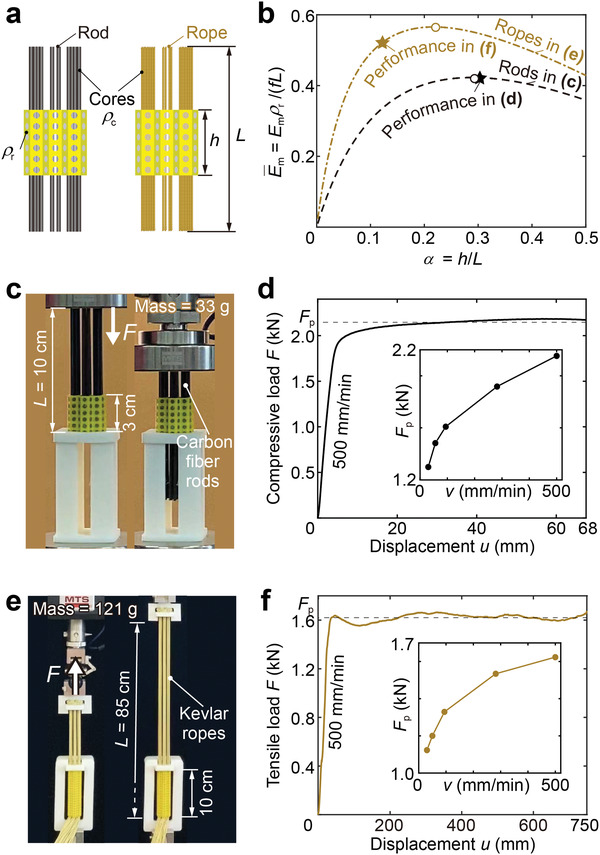
Energy‐absorbing materials with high capacities per unit mass. a) Parameters of the architected materials, using carbon fiber rods or Kevlar ropes as cores, for calculating absorbed energy per unit mass. b) ${\bar{E}}_{m}$‐α curves of the materials with cores sketched in (a) or shown in (c) and (e). Here, *E*_m_ is absorbed energy per unit mass, and the hollow circles represent the maximal values of ${\bar{E}}_{m}$ varying *h*/*L*. The solid stars correspond to the energy‐absorbing performance with parameters shown in (c) and (e). c,d) Architected materials with carbon fiber rods as the cores and the corresponding load‐displacement curve under *h*/*L* = 3/10. e,f) Architected materials with Kevlar ropes as the cores and the corresponding load‐displacement curve under *h*/*L* = 10/85. The insets in (d) and (f) illustrate the dependence of the force plateau *F*_p_ on the loading rate *v*.

Although the above tested architected materials can absorb a large amount of energy, they are heavy since the rods are made of steel. Making use of the high frictional coefficients between silicone elastomers and other materials,^[^ ^57^ ^]^ we replace the steel rods with other light‐weight cores, such as carbon fiber rods and Kevlar ropes, to improve the energy‐absorbing capacity per unit mass of the architected materials. Consider a seven‐column module (**Figure** **4**a) with the height of the reinforced elastomer *h*, mass per unit length of the reinforced elastomer ρ_r_ = 6.6 g cm^−1^, length of the cores *L*, and mass per unit length of the cores ρ_c_ = 1.3 and 0.56 g cm^−1^ for carbon fiber rods and Kevlar ropes, respectively. If the magnitude of the friction force per unit length is *f*, the dissipated energy per unit mass *E*_m_ is:

(2) $$E_{m}=\frac{fh(L-h)}{\rho_{r}h+\rho_{c}L}=\frac{fL}{\rho_{r}}\frac{\alpha (1-\alpha )}{\alpha +\rho_{c}/\rho_{r}}$$

where α≜*h*/*L* is the ratio of the reinforced elastomer height to the core length. For a given core, *E*_m_ monotonically increases with *L*. We define the dimensionless dissipated energy ${\bar{E}}_{m}≜E_{m}\rho_{r}/\left(fL\right)=\alpha \left(1-\alpha \right)/\left(\alpha +\rho_{c}/\rho_{r}\right)$, which only depends on two dimensionless quantities α and ρ_c_/ρ_r_. While ${\bar{E}}_{m}$ monotonically decreases with ρ_c_/ρ_r_, it non‐monotonically increases and then decreases as α increases, reaching the maximal value at an optimal α (Figure 4b).

Two samples were made to demonstrate the practical performance of the design. The first sample (Figure 4c) used 2 mm‐diameter carbon fiber rods as cores. The length of the rods is 10 cm and the height of the reinforced elastomer is 3 cm, so that the length ratio is α = 0.3. The corresponding dimensionless energy‐absorbing quantity ${\bar{E}}_{m}$ approaches the maximal value (see the black dashed line in Figure 4b). This architected material only weights 33 g, but can dissipate energy as large as ≈80 − 140 J, depending on the loading rate (Figure 4d). In other words, the energy‐absorbing capacity reaches ≈2.6 − 4.2 kJ kg^−1^, comparable to the non‐reusable ones.

The second sample adopted Kevlar ropes as cores (Figure 4e). In detail, we used a needle to thread two strands of 1.2 mm‐diameter Kevlar ropes into a 1.8 mm‐diameter elastomer hole. Since the ropes are more compliant, the prestress in this sample is lower than the first one. We enlarged *L* to 850 cm (limited by the working stroke of our testing MTS machine) and *h* to 10 cm to improve *E*_m_. The obtained length ratio is α = 0.12, and the corresponding ${\bar{E}}_{m}$ is about 90% of the maximal value (see the yellow dashed‐dotted line in Figure 4b). The architected material weights 121 g, but can dissipate energy as large as ≈820 − 1190 J, varying with the loading rate (Figure 4f). In other words, the energy‐absorbing capacity is ≈6.8 − 10 kJ kg^−1^, which is even better than the first sample.

## 3 Discussion and Conclusions

**Figure 5:**
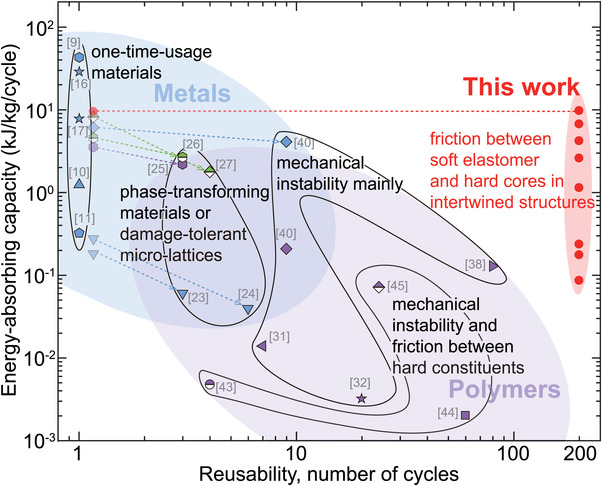
Energy‐absorbing capacity versus reusability of previous and our proposed energy‐absorbing materials. The cycle numbers are the maximum tested loops, and the dotted lines with arrows represent variation of energy‐absorbing capacities with recycle number. Here, all points are calculated by experimental data from references.^[^ ^9^, ^10^, ^11^, ^16^, ^17^, ^23^, ^24^, ^25^, ^26^, ^27^, ^31^, ^32^, ^38^, ^40^, ^43^, ^44^, ^45^ ^]^

We summarize the performance of various energy‐absorbing materials in the literature into **Figure** **5** and Figure S6, Supporting Information. The plot of capacity versus reusability (Figure 5) confirms that the previous materials have trade‐off between capacity and reusability, while the proposed ones (solid red circles) possess both high capacity and high reusability. In a single cycle, the energy‐absorbing capacity of our architected materials is best among the reusable category, reaching $10\;\mathrm{kJ}\;{\mathrm{kg}}^{-1}\text{per}\;\mathrm{cycle}$, which is comparable to the non‐reusable category. Meanwhile, this value remains almost unchanged in 200 tested cycles, while the capacity of traditional reusable designs with relatively large capacity decreases notably in less than ten cycles (dotted lines in Figure 5). If the energy‐absorbing capacity per cycle is multiplied by the number of repeated cycles to calculate the total energy‐absorbing capacity in the entire life of a material, then a conservative estimate of our design is 10 × 200 = 2000 kJ kg^−1^ per life, which is at least 40 times as many as the others (Figure S6, Supporting Information). Actually, this value could be much larger since the designed materials are still intact and reusable after the tested 200 cycles, and there is no sign of notable performance descending.

We attribute the extraordinary performance of the proposed architected materials into two strategies of our design. First, we utilize friction between a soft elastomer and hard constituents rather than between hard particles, which is the strategy of the previous frictional metamaterials. Involving an elastomer facilitates the formation of tightly contacted interfaces with a large true contact area. Second, the intertwined structure strongly anchors the soft elastomer on the stiff frame, which empowers the reinforced elastomer with robust load‐bearing capability and allows larger prestress applied at the frictional interfaces. These interfaces notably improve the capacity of energy‐absorbing architected materials based on friction as shown in Figure 5. In addition, the rate‐dependent frictional behavior of elastomer interfaces endows the reported materials passive adaptability to impacts with different velocities.

The proposed energy‐absorbing architected materials have advantages over the commercial ones on designing reusable energy absorbers, such as fluid‐free vibration dampers for cars.^[^ ^46^ ^]^ Since the energy‐absorbing capacities of our materials are one to two orders of magnitudes higher than the commercial ones, they can save space and weight. Moreover, the load–displacement curve of the proposed material can be adjusted even after manufacturing. In the present design, the elastomer and inserted cores are not bonded, and can be easily disassembled and reassembled. Hence, different energy‐absorbing capacities can be obtained by exchanging the reinforced elastomer or cores, depending on the applications. We believe our design strategy opens a new avenue to obtain high‐performance reusable energy‐absorbing architected materials that can enable novel designs of machines or structures.

## 4 Experimental Section

### Fabrication of the Reinforced Elastomer

The reinforced elastomer samples were manufactured by combining fused deposition modeling (FDM) 3D printing technology (Hori Z300 3D Printer) and mold‐casting process. Materials adopted were commercial FDM 3D printing PLA filaments (eSUN poly lactic acid), bright‐finished 304 stainless steel rods of diameter 1.8 mm and a commercially available silicone elastomer (Dongguan ShinBon New Material Co., Ltd., China). The elastomer consisted of two liquid constituent parts. They were mixed in a ratio of 1:1, then poured into 3D printed molds assembled with PLA frame and steel rods, and finally cured for 6 h at room temperature. These steel rods were then removed after curing, and the corresponding holes left in the elastomer were subsequently inserted with thicker rods/ropes.

### Measurements of the Stress–Strain Curve of the Elastomer

To quantify the mechanical properties of the elastomer, uniaxial tension and compression tests were conducted using a single‐axis MTS testing machine (E44.104). Due to the large deformation and low stiffness of the elastomer, video extensometry, instead of conventional clip‐on extensometry, was used to measure the tensile strain. Specifically, a ruler was placed vertically on one side of the tension specimen, and the coordinates of the markers on the specimen were video recorded (see Figure S5, Supporting Information).

### Experimental Tests of the Proposed Architected Materials

To characterize the energy absorption capacities of the architected materials, uniaxial compression or uniaxial tension tests were performed. The force–displacement curves of the samples were measured under displacement control by a MTS testing machine. The loading rates were from 1  to 500 mm min^−1^, which is the maximal loading speed of the machine. In the experiments, loading platforms, made of PLA, were fabricated by 3D printing.

### Statistical Analysis

All the force–displacement curves were raw data obtained from compression or tension tests. The experimental data of the architected materials and silicone elastomer in Figure 3b–e are presented by means, maxima, and minima. The sample size is included in the figure caption. Statistical analysis was performed with MATLAB software.

### FEM Simulations

In the present studies, several FEM models have been built in commercial software Abaqus/Standard 2014 to estimate the stress distribution in the silicone elastomer under loading. The elastomer was considered as incompressible, and its hyperelastic behavior was formulated by the third order Ogden material model with the strain energy density

(3) $$W\left(\lambda_{1},\lambda_{2},\lambda_{3}\right)=\sum_{i=1}^{3}\frac{2\mu_{i}}{\alpha_{i}^{2}}\left(\lambda_{1}^{\alpha_{i}}+\lambda_{2}^{\alpha_{i}}+\lambda_{3}^{\alpha_{i}}-3\right)$$

where λ_1_, λ_2_, and λ_3_ are principle stretches, and the material parameters were adopted as μ_1_ = 0.322 MPa, α_1_ = 3.248, μ_2_ = 7.949 × 10^−5^ MPa, α_2_ = 13.706, μ_3_ = 6.667 × 10^−2^ MPa, α_3_ = −3.490. These parameters were determined by fitting against experimental data of uniaxial tension and compression tests (for details see Figure S5, Supporting Information).

In addition, Young's Modulus *E* = 3 GPa, Poisson's ratio ν = 0.33, and *E* = 206 GPa, ν = 0.3 were taken for the PLA frame and steel rod, respectively. Eight‐node linear elements with hybrid formulation and reduced integration (C3D8RH) were adopted for the silicone elastomer due to its incompressibility, and eight‐node linear elements with reduced integration (C3D8R) were adopted for the PLA and steel rod. All geometric parameters of the model were consistent with those of the experimental samples.

## Conflict of Interest

The authors declare no conflict of interest.

## Author Contributions

Z.Z. and J.L. designed the research. Z.Z., L.J., and Q.L. supervised the research. J.L. made all of the metamaterial samples, and conducted related experiments and numerical simulations. J.L. and Z.C. measured frictional coefficient between elastomer and steel. All authors discussed the results and revised the manuscript at all stage.

## Supporting information

Supporting Information

Supplemental Movie 1

Supplemental Movie 2

## Data Availability

The data that support the findings of this study are available from the corresponding author upon reasonable request.
