# Supplementary material for: Harnessing Friction in Intertwined Structures for High‐Capacity Reusable Energy‐Absorbing Architected Materials
Source: Adv Sci (Weinh). 2022 Mar 8;9(13):2105769. doi: 10.1002/advs.202105769 (PMC9069190; doi:10.1002/advs.202105769)
Supplement: Supplementary file 1 — Supporting Information [file ADVS-9-2105769-s001.pdf]

Supporting Information

Harnessing Friction in Intertwined Structures for High-capacity Reusable Energy-absorbing Architected Materials

Jinyou Li, Zhe Chen, Qunyang Li, Lihua Jin*, Zhihua Zhao**

Table of Contents

Figure S1. Periodic stiff porous frame of the reinforced elastomer modified from Schwarz primitive minimal surface. 2
Figure S2. Fabrication process of the reinforced elastomer......3
Figure S3. Measurements of rate-dependent μ between the steel and silicone elastomer. 4
Figure S4. FEM model for calculating the nonlinear *N*-*F* relationship of a quarter column......6
Figure S5. Tested mechanical properties of the silicone elastomer and the corresponding Ogden material model. 7
Figure S6. Energy-absorbing capacity per life versus reusability......8
Movies......9
References......9

Figures

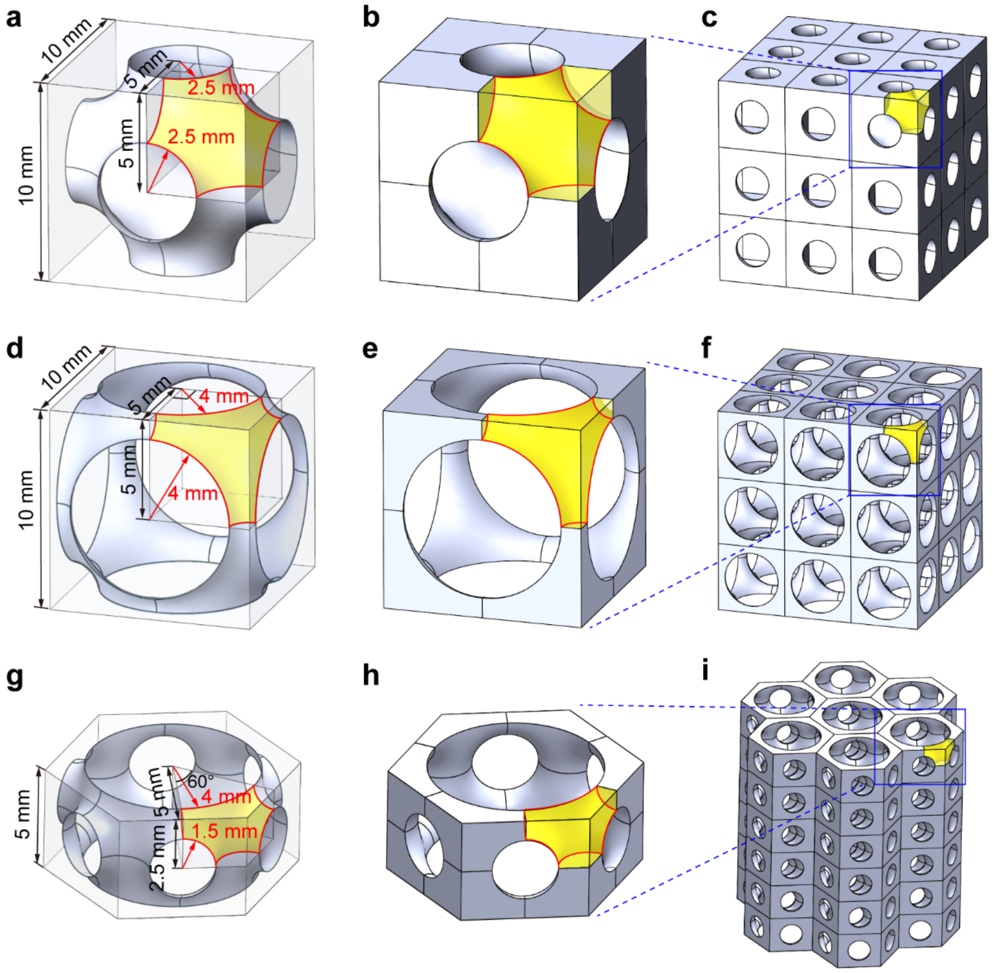

Figure S1. Periodic stiff porous frame of the reinforced elastomer modified from Schwarz primitive minimal surface. a) A cubic unit with a smooth surface modified from Schwarz' P surface. [1] The yellow patch with six red edges is a 1/8 portion of the surface, and each edge is a quarter circle. b) Surface in (a) splits the cube into two domains: inner and outer, and the outer domain is a filled solid. c) 3×3×3 array structure of the cubic cell in (b). d) A cubic unit with a surface modified from (a) by enlarging the circles on the faces of the cube. e) A cubic cell obtained by filling the outer domain in (d) as a solid. f) 3×3×3 array structure of the cubic cell in (e). g) A hexagonal unit with the surface modified from (d) by changing the top and bottom faces from square to hexagon. This modification allows a cell to have more neighbors in the horizontal plane. h) A hexagonal cell obtained by filling the outer domain in (g) by a solid. i) Seven-column array structure of the hexagonal cell in (h), which is the design of the stiff porous frame in the reinforced elastomer.

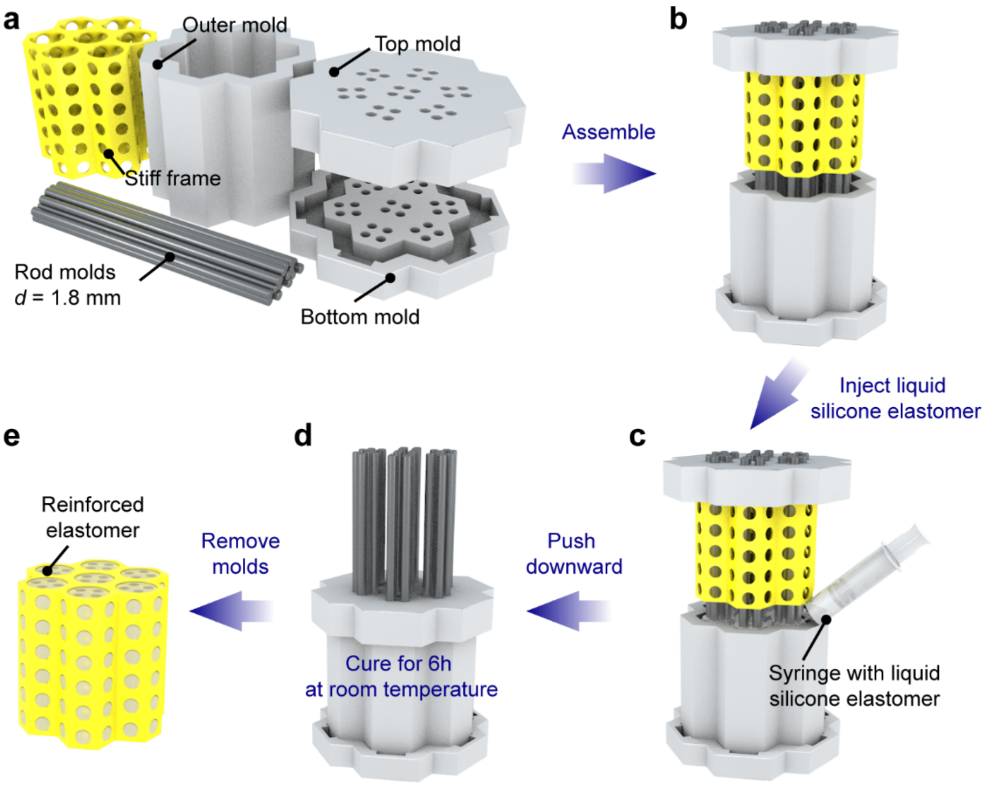

Figure S2. Fabrication process of the reinforced elastomer. a) Components required for manufacturing the reinforced elastomer, including a stiff porous frame and four molds. The stiff frame, outer mold, top mold, and bottom mold are 3D printed through FDM technology (Hori Z300 3D printer) with PLA filaments (eSUN poly lactic acid). The rod molds are commercial 304 stainless steel rods of diameter 1.8 mm. b) Assemble the molds and the stiff frame together. c) Inject pre-cured liquid silicone elastomer with 1:1 ratio of two liquid constituents into the assembled outer mold by a syringe. d) Push the top mold and the stiff frame downward until the top mold encloses the outer mold, and leave the assembly at room temperature for 6 hours to cure the silicone elastomer. e) Remove the molds to get the reinforced elastomer, in which the cured silicone elastomer intertwines with the stiff porous frame.

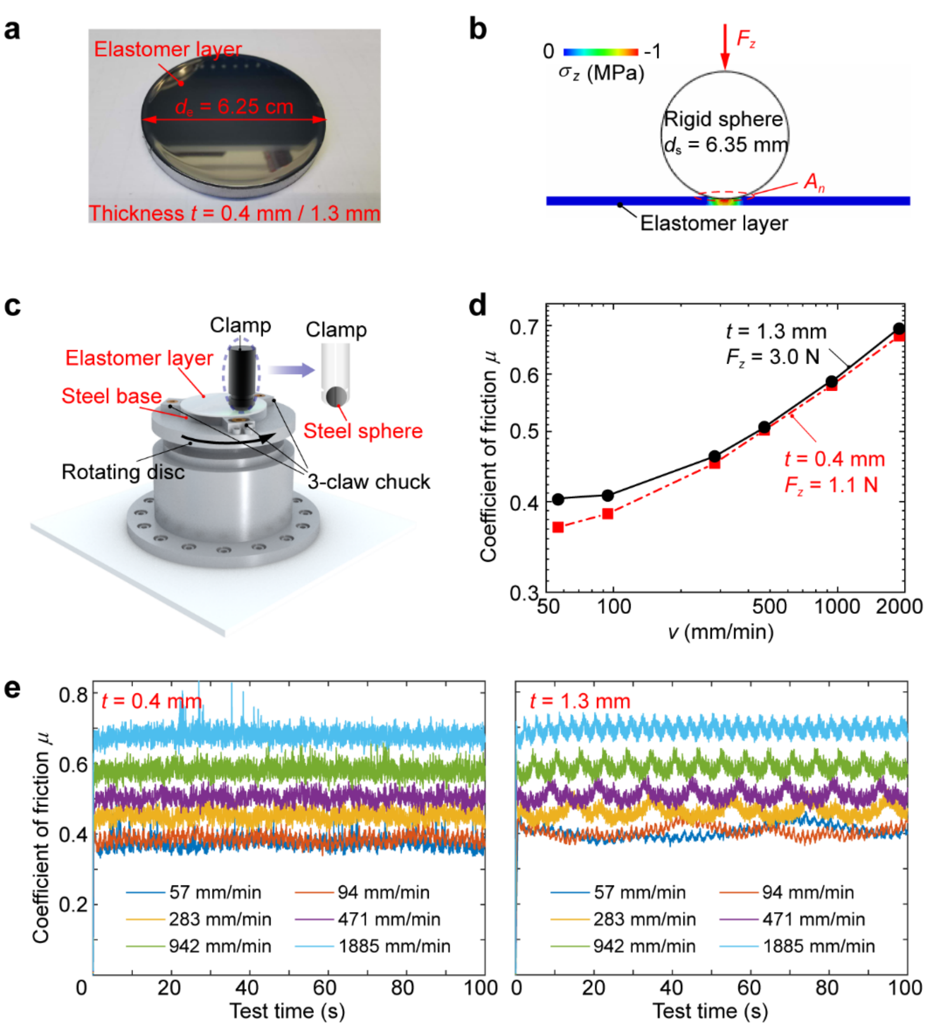

Figure S3. Measurements of rate-dependent μ between the steel and silicone elastomer. a) The specimen of the silicone elastomer layer, with thickness t. b) FEM model to determine the applied force F_z that can maintain the nominal normal stress to be about 0.53 Mpa, which is calculated from Figure S4. c) Schematic of the measurement of μ using a Tribometer (Rtec MFT-V). d) Experimental results of the relationship between μ and the sliding speed v, measured by specimens of thickness 0.4 mm and 1.3 mm. e) Raw data from the tests for the specimens of thickness 0.4 mm and 1.3 mm, respectively.

We use a Tribometer (Rtec MFT-V) to measure the frictional coefficient μ between the steel and silicone elastomer. As shown in Figure S3c, a steel sphere is mounted on the clamp of the machine, and pressed with F_z on the silicone elastomer layer. The steel sphere is a commercial one of diameter 6.35 mm to match the machine, and the silicone layer is cured from liquid form on a steel base of diameter 6.25 cm. We made two elastomer discs with thickness

0.4 mm and 1.3 mm, to match with the varied thickness of the elastomer surrounding a single rod in the proposed architected materials. Since μ depends on normal stress, F_z is applied to maintain the nominal normal stress, defined as F_z divide the contact area A_n, to be about 0.53 MPa. In the experiments, the silicone elastomer layer is driven to rotate at a set speed, while the steel sphere is fixed. The lateral and normal reaction forces are recorded. Calculating their ratio gives μ. Results in Figure S3d indicate that μ increases with the speed from 57 to 1885 mm min^{-1}, covering that in the compression experiments on steel rods in Figure 4 within the speed limit of the MTS testing machine (500 mm min^{-1}).

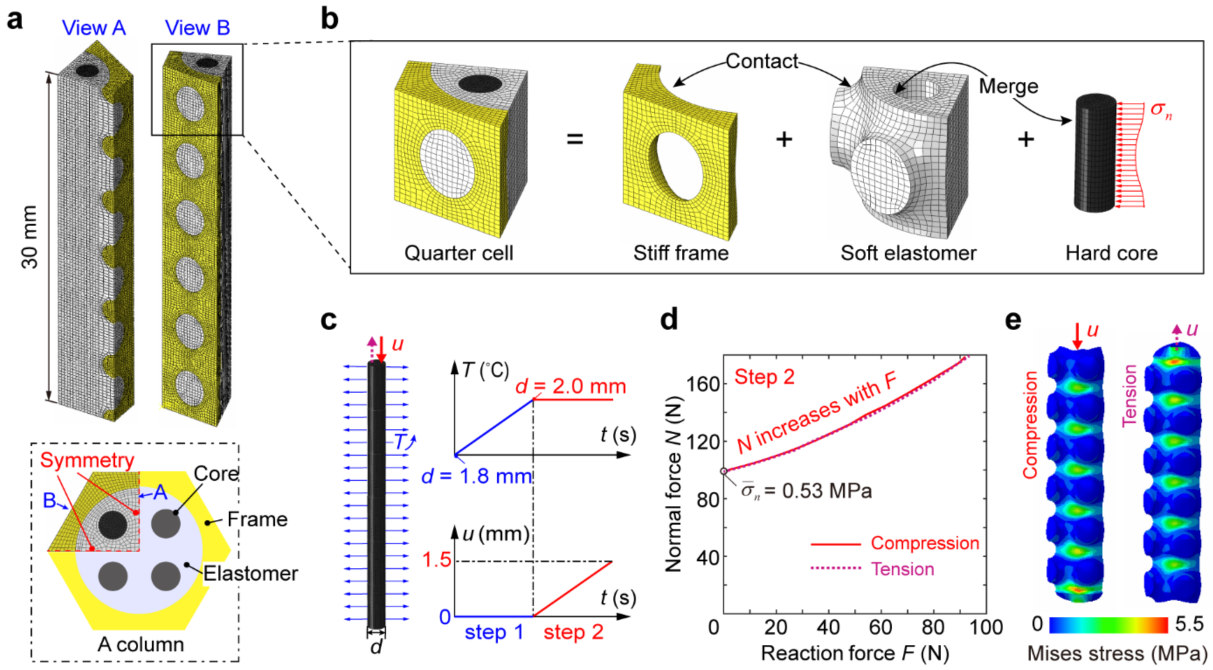

Figure S4. FEM model for calculating the nonlinear *N*-*F* relationship of a quarter column. a) FEM model of a quarter column of the architected material. The red dashed lines in the bottom inset represent symmetry boundary conditions. b) A cell of (a) to illustrate the model in detail. Stiff frame (yellow), silicone elastomer (grey) and hard core (black) are all meshed with eight-node hexahedral elements. The surfaces of the frame and elastomer are set as a contact pair, in which the frictional coefficient is set as 1. The surface nodes of the elastomer and the core are merged together. The constitutive behavior of the silicone elastomer is obtained from Figure S5. Normal force N is calculated as an integration of normal stress σ_n over the core surface. c) Numerical simulations are processed in two quasi-static steps. In step 1, the temperature T in the core is raised until the core diameter increases from 1.8 mm to 2.0 mm to build up prestress in the core and the elastomer. In step 2, the core is forced to move downward or upward, correponding to the compressive or tensive load, by displacement u. The reaction force F is computed. d) Nonlinear *N*-*F* curves for the quarter column calculated in step 2, which coincide for compressive and tensive loads. This is due to the fact that the Young's modules of the core is several orders of magnitude larger than that of the elatomer, so that deformation of the core has negligible effect on the nonlinear feature of the *N*-*F* curve. At the beginning of step 2, the average normal stress $\bar{\sigma}_n$ over the core surface is about 0.53 MPa, which is used to test the rate-dependent frictional coefficient between the steel and the silicone elastomer in Figure S3. e) Mises stress distributions of the elastomer under compressive and tensive loads. They

show no difference under the same load magnitude F = 86 N, which is the maximum load in our tests.

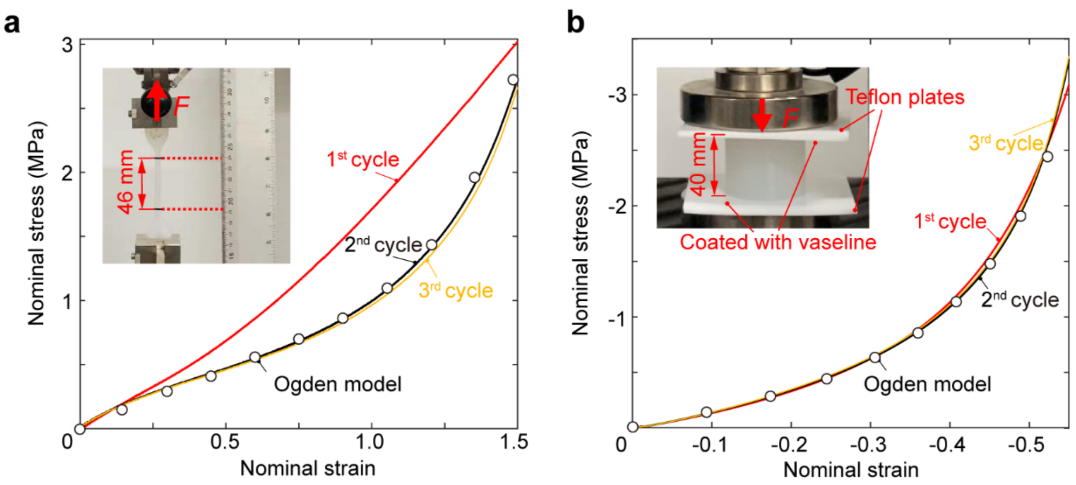

Figure S5. Tested mechanical properties of the silicone elastomer under mutiple cycles and the corresponding Ogden material model. a,b) Stress-strain curve of the silicone elastomer specimen under uniaxial tension and compression tests, and the corresponding theoretical curves of Ogden material model with the parameters in the main context or the next paragraph.

Here, we adopted the third order Ogden material model [2] to formulate the hyperelastic behavior of the elastomer. Its strain energy potential density is

$$W = \sum_{i=1}^{3} \frac{2\mu_i}{\alpha_i^2} \left(\lambda_1^{\alpha_i} + \lambda_2^{\alpha_i} + \lambda_3^{\alpha_i} - 3\right) \tag{1}$$

where λ_1, λ_2 and λ_3 are the principle stretches, and α_i and μ_i (i = 1, 2, 3) are material parameters determined by fitting the experimental data of tension and compression tests. Due to elastomer's Mullins effect, the tensile stress-strain curves change significantly between the first and the subsequent tests. Since our architected materials are for reusing, the second tension curve and the averaged compression curve were adopted as the stress-strain curves for fitting Ogden model. The fitted material parameters are:

$\alpha_1 = 3.248, \alpha_2 = 13.706, \alpha_3 = -3.490,$

$\mu_1 = 0.322$ MPa, $\mu_2 = 7.949 \times 10^{-5}$ MPa, $\mu_3 = 6.667 \times 10^{-2}$ MPa.

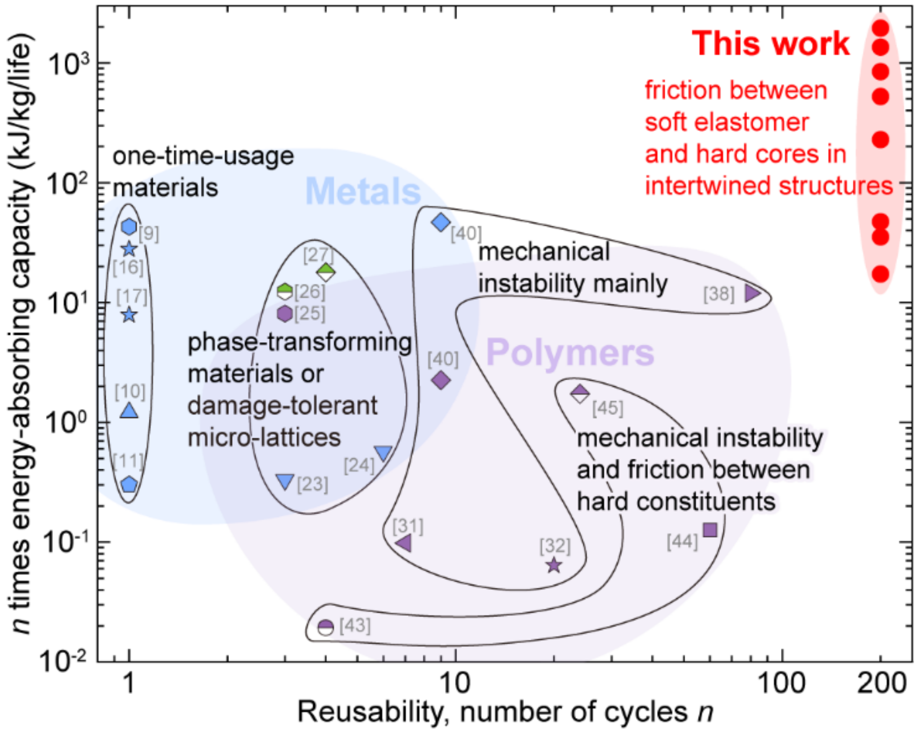

Figure S6. Energy-absorbing capacity per life versus reusability. The numbers *n* of repeated cycles tested in the references are used for the calculation, and the references are listed in the main text.

Movies

Movie S1. Experiments of the architected materials under uniaxial compression or tension.
Movie S2. Experiment of the self-recoverable architected material under uniaxial compression.

References

[1] J. Shin, S. Kim, D. Jeong, H. G. Lee, D. Lee, J. Y. Lim, J. Kim, *Math. Probl. Eng.* **2012**, *2012*, 694194.
[2] J. S. Bergstrom, *Mechanics of Solid Polymers : Theory and Computational Modeling*, William Andrew, San Diego, CA **2015**.
